# The Sunrise Ultraviolet Spectropolarimeter and Imager: Standalone Polarimetric Calibration

**DOI:** 10.1007/s11207-025-02470-8

**Published:** 2025-05-05

**Authors:** F. A. Iglesias, A. Feller, A. Gandorfer, T. L. Riethmüller, A. Korpi-Lagg, S. K. Solanki, Y. Katsukawa, M. Kubo, M. Sánchez Toledo

**Affiliations:** 1 Grupo de Estudios en Heliofísica de Mendoza, CONICET, Universidad de Mendoza, Boulogne Sur Mer 683, 5500 Mendoza, Argentina; 2 Max-Planck-Institut für Sonnensystemforschung, Justus-von-Liebig-Weg 3, 37077 Göttingen, Germany; 3 Department of Computer Science, Aalto University, Konemiehentie 2, 02150 Espoo, Finland; 4 National Astronomical Observatory of Japan, 2-21-1 Osawa, Mitaka, Tokyo 181-8588 Japan

**Keywords:** Instrumentation: polarimeters, Sun: magnetic fields, Sun: UV radiation

## Abstract

Sunrise is a 1-m optical solar observatory carried aloft by a stratospheric balloon. It was developed to study magnetic fields and plasma flows in the solar atmosphere with very high spatial resolution and sensitivity. The Sunrise UV Spectropolarimeter and Imager (SUSI) operates in the 309 – 417 nm range, covering thousands of spectral lines that are poorly accessible from the ground and largely unexplored. The instrument includes a dual-beam polarimeter based on a rotating waveplate, a polarization beam splitter and two custom-made CMOS cameras. SUSI gathers data at high spectral, spatial and temporal resolution. These data are stored onboard during flight. Given that SUSI does not include a polarimetric calibration unit onboard, its polarimetric demodulation matrix is estimated during laboratory calibration measurements prior to its flight. The quality of this calibration is crucial to accurately demodulate the data post-flight and reach the instrument’s maximum polarimetric sensitivity goal of $1\times 10^{-3}$ of the continuum intensity. In this paper, we report the results of eighth polarimetric calibrations of SUSI standalone, acquired at six different wavelengths using artificial LED light sources. The field-dependant demodulation matrices obtained are within the values expected from the design, including their polarimetric efficiencies and temporal stability. The matrices are also confirmed to satisfy the calibration repeatability criterion imposed by the SUSI sensitivity goal.

## The Sunrise Mission

The solar magnetic field is the main driver of a variety of solar phenomena that span a wide range of spatial, temporal, and energetic scales (Solanki, Inhester, and Schüssler 2006). Understanding the occurrence and evolution of these phenomena is important from an astronomical point of view and also from a practical perspective, due to the strong influence the Sun has on Earth’s climate (Gray et al. 2010), space weather and human technology (Zhang et al. 2018). Accurately measuring the magnetic-field interactions with the surrounding plasma at very small spatial scales on the Sun (in the tens of km range), is key to understanding the energy transfer mechanisms occurring at the different layers of its atmosphere (e.g., magnetic reconnection and wave dissipation), and to answering critical open questions in solar physics, see e.g., Borrero et al. (2017).

Sunrise is a solar observatory fed by a 1-m Gregory-type reflector telescope, carried aloft by a zero-pressure stratospheric balloon. The combination of a large aperture, reduced absorption and aberration by Earth’s atmosphere, and state-of-the-art instrumentation, has been essential for the great success of the first two Sunrise science flights in 2009 (Barthol et al. 2011; Solanki et al. 2010) and 2013 (Solanki et al. 2017). For its third science flight, Sunrise III incorporates a new gondola and completely renewed science payload (Korpi-Lagg et al. 2025). Three imaging spectropolarimeters cover the optical spectral range from the infrared (IR) to the ultraviolet (UV). These are the Sunrise Chromospheric Infrared SpectroPolarimeter (SCIP: Katsukawa et al. 2020, 2025), operating in the 765 – 855 nm range; the Tunable Magnetograph (TuMag: Álvarez Herrero et al. 2022; del Toro Iniesta et al. 2025), covering the 517 – 525 nm range; and the Sunrise UV Spectropolarimeter and Imager (SUSI: Feller et al. 2020, 2025) operating in the 309 – 417 nm range. Sunrise image is stabilized by two active closed-loop systems, the gondola pointing system and the Correlating Wave-Front Sensor (CWS). CWS consists of a six-element Shack–Hartmann wave-front sensor, a fast tip-tilt mirror for the compensation of image motion, and an active telescope secondary mirror for focus correction (Berkefeld et al. 2011, 2025).

This instrument suite allows studying small-scale, magnetohydrodynamic phenomena at different heights in the solar atmosphere simultaneously, from the low photosphere to the chromosphere.

## The Sunrise Ultraviolet Spectropolarimeter and Imager

### Instrument Overview

SUSI aims at characterizing a largely unexplored region of the UV solar spectrum ($309-417$ nm), in terms of its polarization properties with high-spatial resolution. The spectral lines present in this range can be used to probe magnetic fields, plasma temperatures and/or velocities at different heights in the solar photosphere and chromosphere (covering a range of about 1300 km). In addition, the high density of spectral lines in the targeted UV range can be used to compensate for the low S/N ratio resulting from the low UV photon flux, by inverting the Stokes profiles of multiple lines simultaneously; see Riethmüller and Solanki (2019) for further details.

**Figure 1:**
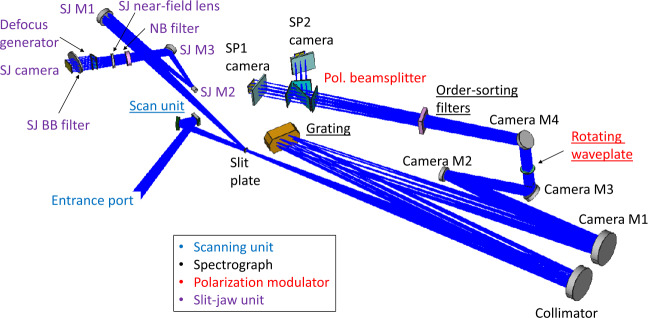
Optical layout of SUSI. We label the elements of the different functional units with different *colors* (see the legend). Underlined components are movable (involving a mechanism). Adapted from Feller et al. (2020).

SUSI is a scanning slit-spectrograph that includes a dual-beam polarimeter, and a separate broad-band channel for context imaging and image restoration. The basic optical layout of SUSI is depicted in Figure 1. The design can be separated into four main functional units, see Feller et al. (2025) for extra details.

**Figure 2:**
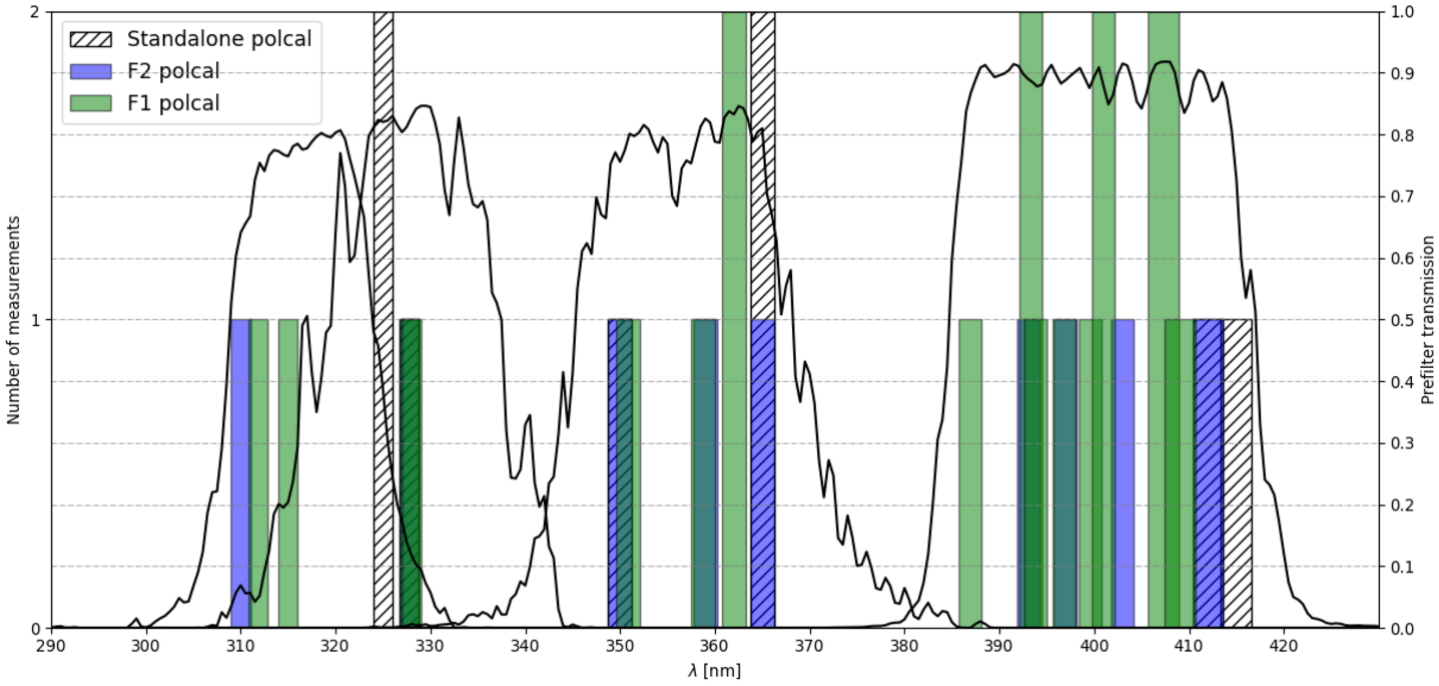
SUSI spectral response and polarimetric calibrations. We show the spectral transmission profiles of the four order-sorting prefilters of the SUSI spectrograph (*continuous black curves*, ref. to the right vertical scale), along with the number of measurements (*height of the vertical bars*, ref. to the left vertical scale) and spectral range (*width of the vertical bars*) of various polarimetric calibrations. The calibrations are performed from different locations in the Sunrise beam path and their spectral coverage may overlap. *Dark green* implies that calibrations at both F1 and F2 were performed. See the legend and the text for further details.

i) Scanning unit, which shifts the solar image (up to $\approx \pm 35$ arcsec) in a direction perpendicular to the spectrograph entrance slit.
ii) Spectrograph (SP), based on a plane-ruled diffraction grating operated in orders 4 to 6, in combination with 4 different order-sorting filters centered at 317, 327, 358, and 401 nm, see Figure 2. The spectral sampling across the SUSI wavelength range varies between about 10 mÅ and 16 mÅ.
iii) Dual-beam polarimeter, formed by a Polarization Modulation Unit (PMU), based on a rotating waveplate, and a polarizing beamsplitter (PBS) that feeds the SP cameras with orthogonal polarization states. The PMU waveplate is a zero-order, air-gapped, double quartz plate with a design retardation 127 deg at 335 nm (chosen as a trade-off between polarimetric efficiency and throughput). The plates have a combined thickness of 1.6 mm, a clear aperture of 39 mm and are hold by AlMgSi1 mounts of diameter 60 mm and thickness 10 mm. The mounts are perforated in the sides to facilitate air circulation in the gap. We verified the retardance variation across the individual plates aperture at wavelengths 625 nm and 594 nm, and room temperature. We found that 97.4% and 99.4% of the components apertures are within $\pm 2$ deg of the mean retardance, for plate one and two respectively. Both surfaces of the waveplate have an anti-reflective coating with less than 0.2% residual reflectivity across the SUSI spectral working range, to suppress fringes and ghost images. The PBS is a 67.4 x 40 x 57.1 mm^3^, 38 mm-clear-aperture, fused-silica prism composed of two halves with inner surfaces cemented at an angle of 55° with respect to the incoming optical axis. The PBS extinction ratios for transmitted and reflected channels are better than 0.1 and 5%, respectively. We measure a practically linear dependence of the PBS reflected channel extinction ratio from 1 to 4% across the FOV covered by SP2 camera in the sensor rows direction. The result of this test, performed using a white light source and the order-sorting prefilter near 355 nm (see Figure 2), is compatible with the angle-dependent extinction at the polarizing beam-splitting surface. For unpolarized light, the relative flux difference between PBS transmitted and reflected beams is below 5%, to balance their photon noise properties. The PMU takes 0.512 s to complete a rotation covering two polarization modulation cycles. One modulation cycle permits the measurement of the four components of the Stokes vector ($S=[I,Q,U,V]$), describing the full polarization state of the incoming beam, independently for each SP camera. The two simultaneous Stokes vectors measured in each SP camera can be combined during data reduction to reduce specific instrumental effects, including motion-induced crosstalk (see e.g., Iglesias and Feller 2019). The goal polarimetric sensitivity depends on the science case and the corresponding data post processing applied (see below), with the lowest limit set to $1\times 10^{-3}$ of the continuum intensity.
iv) Slit-jaw unit, which images the light reflected by the slit plate via a broad-band channel (central wavelength 325 nm, 0.9 nm FWHM) that includes a defocus generator and a single camera. This unit produces two high signal to noise ratio (SNR) images, one focused and one defocused in each half of a single camera. The slit-jaw images are synchronized with the SP cameras allowing for the implementation of post-facto image restoration techniques to reduce residual optical aberrations due to instrumental jitter and seeing, see van Noort (2017).

All three SUSI cameras are custom-built using GSENSE400BSI, back-illuminated CMOS sensors manufactured by Gpixel. This sensor type has a size of 2048 x 2048 px, a 11 $\mu $m pixel pitch, high quantum efficiency (40% at 300 nm and 88% at 400 nm), and very low readout noise (< 1.5 e^−^ at the highest conversion gain). The cameras include a protective 3-mm fused-silica entrance windows with $<1\%$ anti-reflective coating in the 300 – 410 nm wavelength range.

The expected spectropolarimetric performance of SUSI varies across its wide spectral working range due to various instrumental and external effects, including the wavelength-dependent photon flux reaching the cameras. The instrument spatial and temporal samplings are 0.03 arcsec px^−1^ and 256 ms in full-Stokes polarimetric mode, respectively. Note that the final temporal, spatial and spectral (3D) resolutions obtained when imaging an evolving solar signal, depend on the data binning employed to reach the goal SNR (see Iglesias and Feller 2019). SUSI data is stored during flight with the native sampling specified above, allowing for the application of different 3D binning during data reduction and thus different final data properties, depending on the requirements of the science case. Furthermore, the final image quality of the data, which relies on the performance of the Sunrise pointing and CWS systems, and the application of numeric image restoration, strongly affects this SNR vs resolution trade-off. For a more detailed description of SUSI see Feller et al. (2020, 2025).

### Polarization Measurement Scheme

All three SUSI cameras operate at a framerate of 47.040 fps in strict synchronization with the PMU, with an error below $0.36\pm 0.21$ ms. This implies that each modulation cycle (half PMU rotation) is sampled by 12 frames (modulation states) of the SP cameras. The CMOS sensor has a rolling shutter and is exposed continuously with almost perfect duty cycle (99.65%). The time difference between the start of the exposure of two consecutive sensor rows is 10.34 $\mu $s. As a consequence, different sensor rows sample different position angles of the PMU waveplate fast axis ($\theta $). This results in a PMU angular difference between the start of the exposures of rows 1 and 2048 of $\Delta \theta =14.74$ deg.

Given this setup, the intensity level integrated by sensor row $i$ during frame number $k$ can be expressed as:

1 $$ \hat{I}_{i,k} = \Delta t (I_{i}h_{i,k,1}+Q_{i}h_{i,k,2}+U_{i}h_{i,k,3}+V_{i}h_{i,k,4}), $$

where $S_{i}=[I_{i},Q_{i},U_{i},V_{i}]$ is the Stokes vector describing the polarization state of the beam at the entrance port of SUSI, and $h_{i,k}=[h_{i,k,1},h_{i,k,2},h_{i,k,3},h_{i,k,4}]$ is the diattenuation vector averaged during the exposure, namely, the first row of the matrix:

2 $$ M_{i,k}=\frac{1}{\Delta \theta}\int _{\theta _{i,k}}^{\theta _{i,k}+ \Delta \theta} M(\theta ) \delta \theta , $$

where the 4 x 4 Mueller matrix $M(\theta )$ models the polarimetric action of all the optical elements present in the beam path. Note that, we assume the PMU starts each modulation cycle at the same angle ($\theta _{0,0}$) and rotates at a constant angular speed $\omega $, so that $\theta _{i,k}=\omega [t_{k}+(i-1)*10.34~\mu s] + \theta _{0,0}$, where $t_{k}$ is the starting time of frame $k$ exposure. In addition, we assume $S$ does not change during the full modulation cycle (256 ms, half PMU rotation). Using Equation 1, we can express the 12 integrated intensities at sensor row $i$ during a complete modulation cycle in matrix notation:

3 $$\begin{gathered} \begin{bmatrix} \hat{I}_{i,1} \\ \hat{I}_{i,2} \\ \vdots \\ \hat{I}_{i,12} \end{bmatrix} = \begin{bmatrix} h_{i,1,1} & h_{i,1,2} & h_{i,1,3} & h_{i,1,4} \\ h_{i,2,1} & h_{i,2,2} & h_{i,2,3} & h_{i,2,4} \\ ... & & & \\ h_{i,12,1} & h_{i,12,2} & h_{i,12,3} & h_{i,12,4} \end{bmatrix} \begin{bmatrix} I_{i} \\ Q_{i} \\ U_{i} \\ V_{i} \end{bmatrix}, \end{gathered}$$

4 $$\begin{gathered} \hat{I}_{i} = O_{i} S_{i}, \end{gathered}$$

where $O_{i}$ is the 12 x 4 modulation matrix corresponding to sensor row $i$. Note that, we have used a notation that makes the dependence of $O$ on $i$ explicit, to account for the effect of the rolling shutter and the PMU rotating waveplate. However, there is an additional implicit variation of $O$ across the sensor FOV, due to the variation of the optical properties of the elements in the beam path across their clear aperture, see Section 3.2.

An estimation of the input Stokes vector is retrieved from the registered intensities after the Sunrise flight, during data reduction, by doing:

5 $$ \hat{S_{i}}=\hat{O}^{-1}_{i}\hat{I}_{i}=\hat{D}_{i}\hat{I}_{i}, $$

were $\hat{O_{i}}$, and its pseudo-inverse, the demodulation matrix $\hat{D_{i}}$, are estimations of the true matrices and obtained during the SUSI polarimetric calibration procedure, see Section 3. An analogous analysis can be done for the SP2 camera.

To facilitate the development of the polarimetric calibration and data reduction routines before the SUSI final integration, and to assist in evaluating the instrument performance during verification, we developed a numeric simulation code named SUSIM. SUSIM models only the polarimetric aspects of SUSI by computing an approximation of $M_{i,k}$ (Equation 2) using the theoretical Mueller matrices of all the optical components in the beam path. The matrices required to model all the SUSI elements are those of mirrors, partial linear polarizers, retarders, or a combination of them, see e.g., Stenflo (1994) for their detailed description. The input physical parameters required by the models (retardances, reflexivities, transmissions, position angles, etc.) were obtained from design and/or manufacturer specifications, catalogues and/or custom laboratory measurements. SUSIM also incorporates a simple camera model, including sensor bias, conversion gain, noise and non-linearity. All the SUSIM input model parameters can be set to vary across the FOV to study the polarimetric effects of various instrumental artifacts. We note that the values of some of the input parameters can differ considerably from the real ones, and that custom laboratory measurements and model validation were not carried out on all individual components due to laboratory (e.g., short wavelength) and operative constraints. Thus, we empathize that SUSIM is not used during the calibration procedure described in Section 3.1, nor as a ground reference, only as guideline for integration and functional verification.

## SUSI Standalone Polarimetric Calibration

### Laboratory Setup and Measurements

**Figure 3:**
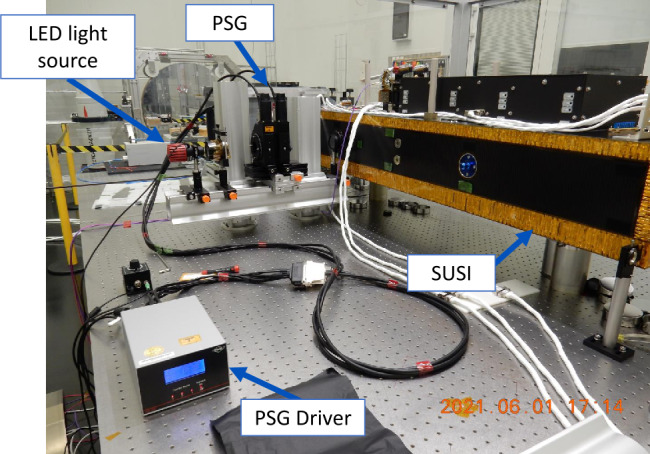
SUSI during a laboratory polarimetric calibration, with the polarization state generator (PSG) located in front of the SUSI entrance port. See the text for extra details.

An accurate calibration of the SUSI polarimetric response is crucial to reach the desired magnetic sensitivity. Since there is no polarization state generator (PSG) onboard, SUSI polarimetric calibration is done on the ground before flight, see Figure 3.

The calibration procedure requires generating a set of known polarization states with a laboratory PSG, that are measured with SUSI to derive an estimated modulation matrix, $\hat{O}$, for each SP camera and sensor pixel (see Equation 5). All the optical elements in between the PSG and the SP cameras are accounted for in the calibration. Note that the polarimetric response of the full beam path, from Sunrise entrance aperture to SUSI SP cameras, is required to retrieve reliable solar quantities. This end-to-end polarimetric calibration has been split into partial calibrations, done by locating the PSG at the Sunrise primary (F1) and secondary (F2) foci, and at the entrance port of SUSI, see Figure 3. Here we report only the results of the latter. The end-to-end calibration will be published in a follow up paper, see end of Section 4.

The illumination unit employed is formed by a LED plus two lenses that project the LED radiative surface to the SUSI slit plate with the nominal focal ratio of F/25, to ensure flight-representative illumination of all optical components within SUSI. The unit illuminates the PSG, composed of a linear polarizer with fast optical axis at position angle $\phi $, and a waveplate with fast axis at position angle $\alpha $ and retardance $\delta $, mounted in two independent motorized rotational stages. Following Equation 4, and dropping the row index $i$ for simplicity, the modulated intensities measured for PSG polarization state $l$ can be expressed as:

6 $$ \hat{I}_{l} = I_{{\mathrm{PSG}}, l}O M_{{\mathrm{PSG}}}(\phi _{l},\alpha _{l}, \delta _{l}) \begin{bmatrix} 1&0&0&0 \end{bmatrix} ^{T}, $$

where $M_{{\mathrm{PSG}}}(\phi _{l},\alpha _{l},\delta _{l})$ is the normalized Mueller matrix of the PSG, $T$ denotes the transpose, and $I_{{\mathrm{PSG}},l}$ is the PSG output intensity. Note that we assume the LED source to be unpolarized, which is not likely the case. However, our model accounts not only for a variable LED intensity, but also for its polarization via the variable term $I_{{\mathrm{PSG}},l}$ because the LED’s polarization only affects the PSG output intensity and not its normalized polarization values. A total of 40 different polarization states are generated during each calibration by changing the PSG position angles, $\phi _{l}$ and $\alpha _{l}$, via the PSG motor driver, see Figure 3. We use 10 values of $\alpha _{l}=\{0,36,72,\ldots,324\}$ deg for each of the 4 values of $\phi _{l}=\{0,90,135,225\}$ deg. The value $\phi =0$ is aligned with the Sunrise Y-axis (cf. Korpi-Lagg et al. 2025 for a definition of the Sunrise coordinate system), which is in elevation direction (perpendicular to the horizon) on the Sky, and defines the $+Q$ direction. We consider $\delta _{l}$ changes with $l$ for a given pixel due to the combination of the PSG waveplate rotation and a variation of $\delta $ across the waveplate clear aperture. The properties of the PSG waveplate are not exactly known. Measuring $\delta $ for all the wavelengths of interest and across the waveplate clear aperture with error $<5\%$ is difficult. Likewise, the rotational stages mounting accuracy limit, plus the error when determining $\alpha $ for all the wavelengths of interest, result in $\sim 1$ deg errors.

The 40 calibration measurements (Equation 6) form an over-determined system of non-linear equations. For each pixel in the FOV, there are 480 (40 x 12) measured intensity values and 98 unknowns, namely, the 48 elements of the modulation matrix, $O$; one constant error in $\alpha _{l}$ (denoted $\Delta \alpha $); 10 errors in $\delta _{l}$, one per PSG waveplate position (denoted $\Delta \delta _{l/10}$); and the 39 values of $I_{{\mathrm{PSG}},l}$ normalized to the first measurement, denoted $\kappa _{l}$. We solve this system of equations using a Levenberg–Marquardt, least-squares fit that minimizes the following merit function:

7 $$ \sum _{l=1}^{40}\Big|\Big| \hat{I}_{l} - \hat{O}\,M_{{\mathrm{PSG}}}(\phi _{l}, \alpha _{l}+\Delta \alpha ,\delta _{l}+\Delta \delta _{l/10}) \begin{bmatrix} \kappa _{l}\,I_{{\mathrm{LED}}, 0}&0&0&0 \end{bmatrix} ^{T} \Big|\Big|^{2}, $$

for free parameters $\hat{O}$, $\Delta \alpha $, $\Delta \delta _{l/10}$ and $\kappa _{l}$. As initial conditions we use $\Delta \alpha =0$, $\Delta \delta _{l/10}=0$, $\kappa _{l}=1$ and the $\hat{O}$ obtained after assuming $M_{{\mathrm{PSG}}}$ is perfectly known, i.e. the linear problem described by Collados (1999) is solved for each sensor row $i$ after averaging all columns. The values of $\phi _{l}$ and $\alpha _{l}$ are obtained from laboratory measurements and the angles commanded to the rotational stages. $\delta _{l}$ is taken from the manufacturer specifications, the PSG retarder is a 40-mm, two-plate (quartz and MgF_2_), cemented achromatic waveplate. $\delta _{l}$, $\phi _{l}$ and $\alpha _{l}$ are assumed constant across the PSG clear aperture. To reduce computation time and the influence of noise, the images are binned in the columns direction every 30 px before fitting.

SUSI standalone polarimetric calibrations were carried out during 2021 at the integration facilities of the Max Planck Institute for Solar System Research. The main specifications of the eight independent calibration measurements are shown in Table 1. Note that different integration times were used to account for the various signals levels obtained at different wavelengths. Additionally, limitations in the light source and/or related feeding optics did not allow the illumination of the full imaging sensors, thus, we specify the region of interest (ROI) utilized.

**Table 1:** List of SUSI standalone polarimetric calibrations. All measurements were acquired with the same optical setup, except for the LED light source (see table notes), during 2021. The integration time specified in column No. 5 corresponds to each of the 40 different PSG configurations, while the number of frames (expressed in thousands of frames) is the total acquired. The mean temperature given in column No. 6 is that of the PMU and has a Root Mean Squared (RMS) stability of . Column No. 7 indicates the overall mean signal for each SP camera (SP1;SP2). Dark data were acquired immediately after each calibration.

ID	Date mm.dd	Spect. range [nm]	ROI [px^2^]	Integration [kfrm]; [min]	Temp. [^∘^C]	Signal [DN]
325u	07.06	324.00 – 326.00	900 × 1920	144; 1.3	22.66	8.8;7.0^(1)^
325d	07.06	324.00 – 326.00	780 × 1920	144; 1.3	22.58	5.5;4.0^(1)^
327	06.10	326.81 – 328.80	1100 × 1920	144; 1.3	22.60	11.1;8.7^(1)^
350	06.09	348.73 – 351.27	1000 × 1920	48; 0.43	22.65	134.3;95.0^(2)^
365a	06.03	363.76 – 366.25	1770 × 1920	48; 0.43	22.64	348.6;292.4^(2)^
365b	06.08	363.76 – 366.25	1770 × 1920	48; 0.43	22.60	346.7;292.1^(2)^
412	07.05	410.37 – 413.63	1750 × 1920	48; 0.42	22.53	576.2;438.7^(3)^
415	06.08	413.38 – 416.62	1750 × 1920	48; 0.43	22.64	581.6;421.1^(3)^

^(1)^LED model M325L5. ^(2)^LED model M365LP1. ^(3)^LED model M415LP1.

### Results

**Figure 4:**
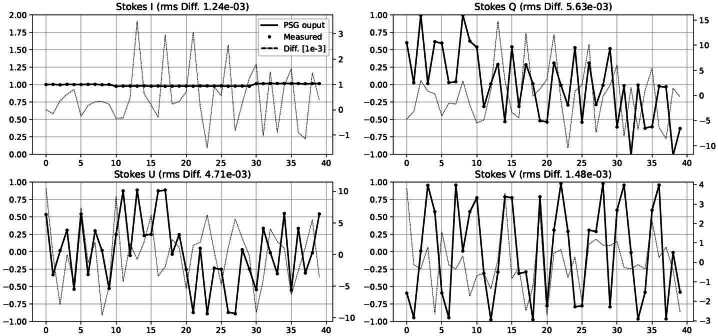
Spatial mean of the 40 Stokes parameters measured during calibration 365b, see Table 1. We show the PSG output (*continuous line*), the Stokes parameters measured by camera SP1 (*circles*), and their difference (*dotted line*, ref. to the right scale in fractions of $10^{-3}$).

**Figure 5:**
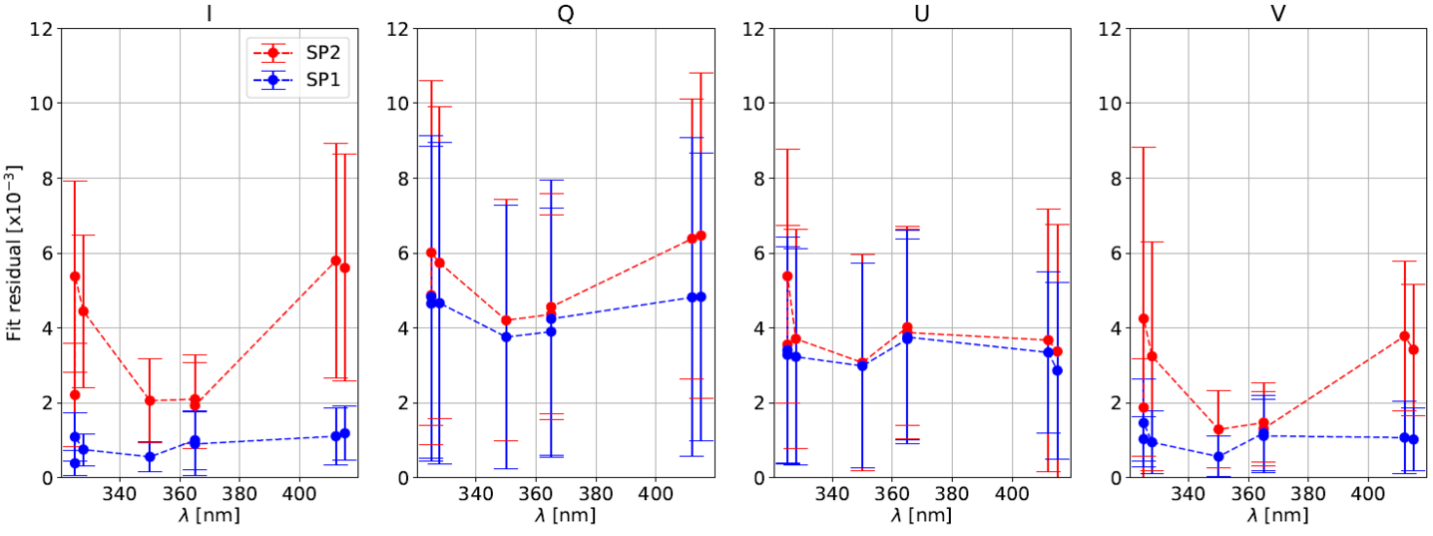
Mean (*dots*) and RMS (*error bars*) fit Stokes errors (ref. to Figure 4) for all standalone calibration measurements detailed in Table 1, and both SP cameras (see legend). Note that the vertical scale is in fractions of $10^{-3}$.

The spatially averaged difference between the 40 Stokes parameters measured during calibration (Equation 5) and those produced by the fit PSG (Equation 7) is presented in Figure 4 for measurement 365a. The errors in the normalized Stokes parameters are in the $10^{-2}$ to $10^{-3}$ range, typical for solar polarimeters calibrations, and within the requirements of SUSI (see Section 3.3). Figure 5 summarizes these Stokes errors for all standalone calibration measurements by specifying their mean and RMS computed over the 40 parameters. Note that the calibrations of the SP2 camera have generally larger errors, which can be attributed to the worse performance (transmission, extinction ratio, etc.) of the PBS reflected beam. This translates into a lower flux reaching camera SP2, see Table 1, and a stronger variation of the polarimetric response across SP2 FOV, among others (see below).

**Figure 6:**
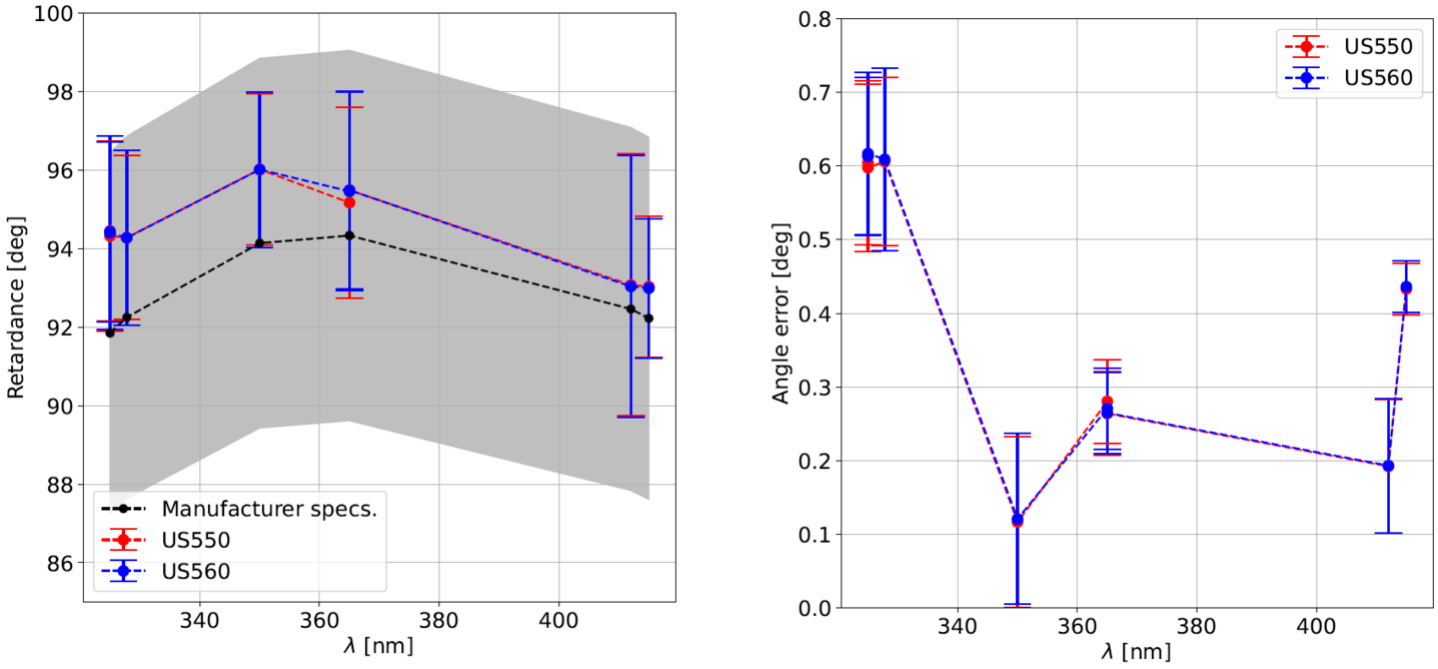
Fit PSG retarder properties vs. wavelength. We show the mean (*dots*) and RMS (*error bars*) retardance (*left panel*) and position angle error (*right panel*) of the PSG retarder for both SP cameras (see legend). The mean and RMS of the retardance include all 10 maps. We also detail the retardance specified by the manufacturer, including its nominal value (*black dots*) and tolerance (*gray band*).

The fitted PSG retarder parameters for all calibrations are summarized in Figure 6. The wavelength dependence of the position angle error $\Delta \alpha $ is $<0.65^{\circ}$, and practically identical for both cameras, even though the fits are independently done. Likewise, the mean and RMS retardance $\Delta \delta _{l/10}$, computed spatially and across the 10 maps, is practicaly identical for both cameras, and lies within the tolerance specified by the manufacturer.

**Figure 7:**
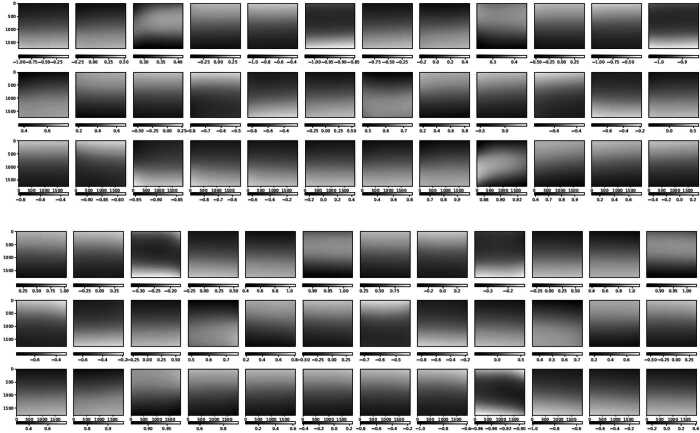
Modulation matrices fitted to measurement 365a for cameras SP1 (*upper panel*) and SP2 (*lower panel*). The vertical and horizontal directions correspond to the spatial (along the spectrograph slit) and spectral dimensions, respectively. We present the transposed matrix with each row normalized to its first element, thus the first column is not shown.

**Figure 8:**
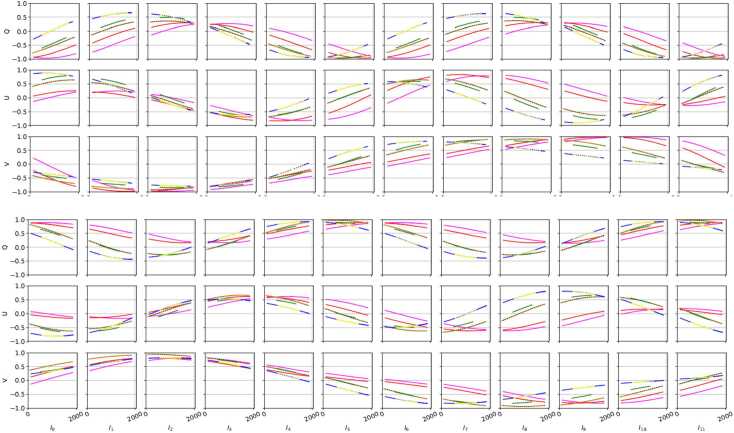
Average along the spectral dimension of the standalone SUSI modulation matrices (ref. to Figure 7) fit to measurements 415 (*magenta*), 412 (*red*), 365a (*black*), 365b (*orange*), 350 (*green*), 327 (*yellow dotted*), 325u (*blue*), and 325d (*blue*).

The modulation matrices fitted to measurement 365a, with each row normalized to its first element, are shown in Figure 7. This normalization forces the column-wise sum of the associated demodulation matrix to be zero, which implies that the case of all-equal modulated intensities corresponds to zero input polarization. Note that the vertical (spatial dimension) gradient, produced by the rolling shutter, practically dominates the matrices variations across the FOV. This gradient is evidenced in Figure 8, that presents the average in the spectral dimension (horizontal) of the modulation matrices fit to all the calibration measurements in Table 1. Given the cameras’ optimal duty cycle, the row variation of the matrix elements along a given column in Figure 8 represents the polarimetric modulation curve. The latter changes its peak-to-peak amplitude according to the wavelength dependence of the polarimetric efficiencies (see below). Other variations of the polarimetric response across the SUSI FOV, not due to the rolling shutter, are discussed in Section 3.3.

**Figure 9:**
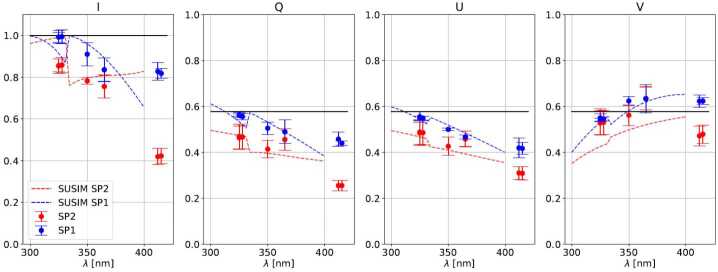
Measured (*dots*), modelled (*dashed lines*) and ideally balanced (*black lines*) polarimetric efficiencies vs. wavelength for each camera, see the legend. For the measured values we show the mean (*dots*) and 3 times the RMS (*error bars*) computed over the sensor area. See the text for extra details.

The polarimetric efficiencies quantify the photometric noise propagation to the demodulated Stokes images, see e.g., Collados (1999). Figure 9 shows the mean efficiencies derived for all calibration measurements, along with the values corresponding to an ideal, perfectly-balanced polarimeter, and the design efficiencies derived using SUSIM (see end of Section 2.2). It can be seen that the measured efficiencies are within $\approx 10\%$ of the design values, with the reflected PBS channel (SP2) presenting up to $\approx 20\%$ lower efficiencies in Q, U and V. In the latter, SP2 deviates ($\approx 10\%$) for shorter wavelengths with respect to the design behavior, which is likely related to the larger fit residuals found for this camera (see Figure 5). Efficiencies in Q and U are larger for shorter wavelengths by design, where the expected SNR values are low due to the lower photon flux, instrument transmission and transversal solar magnetic fields.

### Calibration Stability

Temporal, spectral and spatial variations of the instrument response limit its polarimetric accuracy, because the calibration can not be done at the exact same configuration used for solar data acquisition during the Sunrise flight. We define an upper limit for the acceptable multiplicative errors due to these variations using the Stokes crosstalk matrix defined in Ichimoto et al. (2008), namely:

8 $$ |\Delta X_{{\mathrm{max}}}| = \begin{bmatrix} - & a/p_{l}& a/p_{l}& a/p_{l} \\ \epsilon & a & \epsilon /p_{l}& \epsilon /p_{c} \\ \epsilon & \epsilon /p_{l} &a & \epsilon /p_{c} \\ \epsilon & \epsilon /p_{l} & \epsilon /p_{l} & a \end{bmatrix} , $$

where $p_{l}$, $p_{c}$, $\epsilon $, and $a$ are the expected maximum linear and circular polarization levels, the required polarimetric sensitivity, and the scale error, respectively. Note that, an error of the first element ($I\to I$) is not specified because it represents the system transmission which is not determined by the polarimetric calibration. As the most demanding SUSI observing regime we set $\epsilon =1\times 10^{-3}$, see Section 2.1, and $a=0.05$, corresponding to a $\approx 5\%$ error in the retrieved magnetic field using traditional inversion techniques (Quintero Noda et al. 2017). In addition, we consider the solar linear signals to be low compared to the linear instrumental polarization, thus select a pessimistic value for the latter $p_{l}=0.15$; and adopt an upper limit of $p_{c}=0.2$ due to solar circular signals expected outside of sunspots, i.e., quiet Sun or small pores, where the aimed sensitivity is $\epsilon $. For further information on these estimations see Riethmüller and Solanki (2019) and Feller et al. (2025). Given these values, we get a maximum error matrix of:

9 $$ |\Delta X_{{\mathrm{max}}}| = \begin{bmatrix} - & 330 & 330 & 330 \\ 1.0 & 50 & 6.7 & 5.0 \\ 1.0 & 6.7 & 50 & 5.0 \\ 1.0 & 6.7 & 6.7 & 50 \end{bmatrix} \times 10^{-3}. $$

Equation 9 defines the maximum acceptable crosstalk produced by a non-calibrated variation of the instrument modulation matrix. If such a variation is modeled by matrices $\hat{O}_{a}$ and $\hat{O}_{b}$, the resulting error can be estimated by:

10 $$ |\Delta X_{a,b}|=|\hat{O}^{-1}_{a}\hat{O}_{b}-\mathbf{1}|, $$

where $\mathbf{1}$ is the identity matrix and the modulation matrices are normalized to their first element.

#### Temporal and Spectral Variation

The errors due to the temporal instability of the instrument polarimetric response can be estimated by comparing the modulation matrices obtained at the same wavelengths but different times. For measurements 365a and 365b, acquired five days apart, we get for each SP camera:

11 $$ |\Delta X_{365a,365b}|_{\mathrm{SP1}} = \begin{bmatrix} -&0.2\pm 0.2&0.2\pm 0.3&0.9\pm 0.5 \\ 0.3\pm 0.1&0.8\pm 0.2&2.1\pm 0.6&1.2\pm 0.2 \\ 0.5\pm 0.1&1.9\pm 0.5&0.6\pm 0.2&0.5\pm 0.3 \\ 0.3\pm 0.1&1.8\pm 1.4&0.5\pm 0.1&0.9\pm 0.2& \end{bmatrix}\times 10^{-3}, $$

12 $$ |\Delta X_{365a,365b}|_{\mathrm{SP2}} = \begin{bmatrix} -&0.7\pm 0.7&0.7\pm 0.6&1.6\pm 0.6 \\ 0.2\pm 0.5&0.2\pm 0.6&2.4\pm 0.6&1.3\pm 0.4 \\ 0.7\pm 0.5&2.8\pm 0.5&0.1\pm 0.5&0.0\pm 0.5 \\ 0.3\pm 0.2&1.2\pm 1.4&0.1\pm 0.4&0.2\pm 0.4 \end{bmatrix} \times 10^{-3}, $$

where each element indicates the mean ± the RMS computed over the sensor area. Note that the mean values of all elements in both channels are smaller than $|\Delta X_{{\mathrm{max}}}|$. Thus, these calibrations satisfy the stability requirements.

A similar analysis on temporal stability, combined with wavelength dependence of the calibrations, can be done by comparing the overlapping FOV of the modulation matrices obtained in the 325u, 325d and 327 measurements. The results are:

13 $$ |\Delta X_{325u,327}|_{\mathrm{SP1}} = \begin{bmatrix} -&2.9\pm 2.5&0.8\pm 1.1&0.1\pm 1.8 \\ \mathbf{{1.1\pm 2.4}}&3.0\pm 1.8&2.4\pm 2.3&0.5\pm 1.1 \\ 0.5\pm 1.2&1.3\pm 1.6&0.7\pm 1.2&0.4\pm 1.1 \\ 0.3\pm 1.4&0.7\pm 1.0&0.3\pm 0.6&1.3\pm 2.4& \end{bmatrix}\times 10^{-3}, $$

14 $$ |\Delta X_{325u,327}|_{\mathrm{SP2}} = \begin{bmatrix} -&3.8\pm 1.0&1.9\pm 2.2&0.6\pm 1.3 \\ \mathbf{{1.8\pm 0.5}}&1.4\pm 4.4&2.7\pm 1.6&0.0\pm 1.5 \\ 0.0\pm 0.4&1.6\pm 1.2&3.6\pm 3.2&0.2\pm 2.8 \\ 0.1\pm 0.4&1.0\pm 1.1&0.6\pm 0.5&3.0\pm 2.1 \end{bmatrix} \times 10^{-3}, $$

and,

15 $$ |\Delta X_{325d,327}|_{\mathrm{SP1}} = \begin{bmatrix} -&1.5\pm 1.0&0.2\pm 1.2&1.2\pm 2.6 \\ 0.4\pm 2.2&1.5\pm 1.8&2.1\pm 2.0&0.3\pm 3.3 \\ {0.9\pm 2.1}&1.9\pm 2.3&0.7\pm 1.6&1.2\pm 0.6 \\ 0.1\pm 1.1&2.4\pm 2.2&1.1\pm 1.0&0.8\pm 1.9 \end{bmatrix} \times 10^{-3}, $$

16 $$ |\Delta X_{325d,327}|_{\mathrm{SP2}} = \begin{bmatrix} -&1.9\pm 1.7&1.3\pm 1.5&2.3\pm 2.3 \\ 0.3\pm 0.9&3.8\pm 5.3&1.5\pm 2.0&0.1\pm 1.2 \\ {0.6\pm 0.6}&1.7\pm 1.6&1.8\pm 4.0&0.5\pm 2.6 \\ {0.6\pm 1.0}&3.3\pm 1.2&1.9\pm 0.6&3.3\pm 3.7& \end{bmatrix}\times 10^{-3}, $$

which are smaller than $|\Delta X_{{\mathrm{max}}}|$, except for the elements in the first column highlighted in bold. The latter is discussed in Section 3.3.3. These results show that the polarimetric response of SUSI alone is stable within limits for measurements taken approximately a month apart. One month is the period of time between the final pre-flight polarimetric calibration and the flight. Note that the PMU is thermally stabilized at  during the calibration measurements, see Table 1. The PMU is also thermally stabilized during flight and expected to be within  with respect to this mean calibration temperature. Using the thermal constant specified by the manufacturer at the design wavelength, $-8\times 10^{-5}$, we can estimate an error matrix produced by the waveplate retardance variation due to the temperature difference between calibration and flight conditions. Using a more conservative thermal difference of , all terms in the error matrix are smaller than $8\times 10^{-4}$, and thus below $|\Delta X_{{\mathrm{max}}}|$. We note that this analysis does not consider the thermal effect on the optical axes clocking of the two plates forming the PMU waveplate, see Harrington et al. (2020). This may introduce further calibration errors that will be tackled during the planned residual crosstalk correction (see Section 3.3.3). Finally, these results show that the modulation matrix is sufficiently achromatic in the $326\pm 1$ nm range. Nonetheless, we have acquired ground polarimetric calibrations centered at exactly the same wavelengths of practically all planned SUSI observations, see Korpi-Lagg et al. (2025).

#### Variation Across the FOV

**Figure 10:**
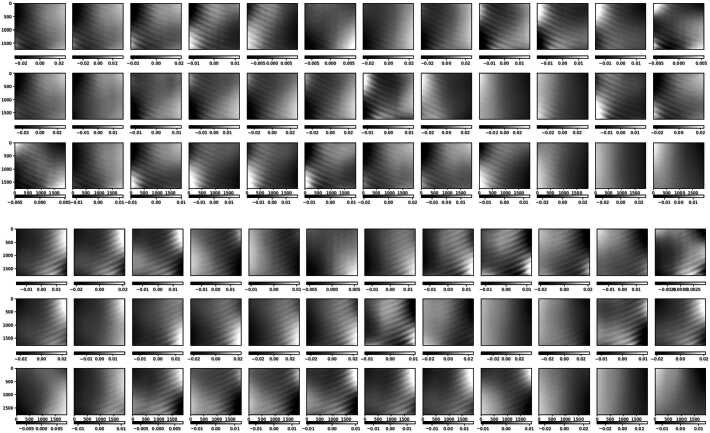
Same as Figure 7, except that the average in the spectral dimension (ref. to Figure 8) was subtracted from each image.

**Figure 11:**
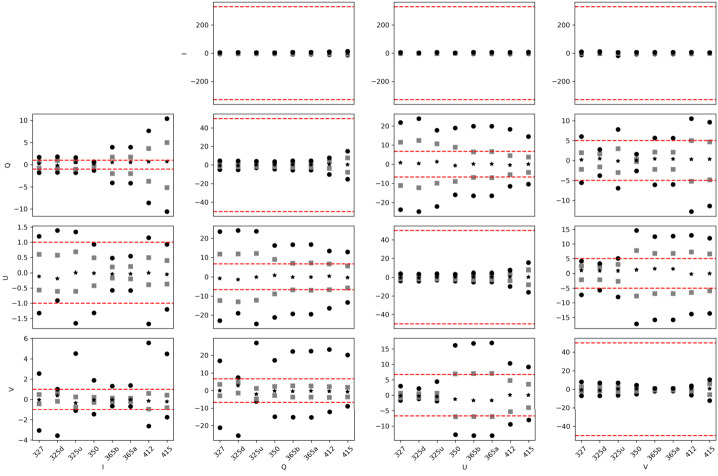
Estimated crosstalk that would be produced by the spatial variations of the modulation matrix if left uncorrected. We show the percentiles 99 and 1 (*black circles*), 25 and 75 (*grey squares*) and the median (*black asterisk*) for each element of $|\Delta X_{\mathrm{sp\_avg}}|$ and each calibration measurement (*horizontal axes*) of the SP1 channel. The *red lines* denote the limits defined by $|\Delta X_{{\mathrm{max}}}|$.

There are three effects producing the main observed variations of the SUSI modulation matrices across the FOV. Firstly, the rolling shutter effect discussed in Section 2.2, which clearly dominates Figure 7. Secondly, the intrinsic variation of the SUSI polarimetric response, produced by the change of the polarization properties across the clear aperture of the optical components in between the PSG and the PMU. This intrinsic variation is visible in Figure 10, which shows the same as Figure 7 except that the average in the spectral dimension ($\hat{O}_{\mathrm{sp\_avg}}$, ref. to Figure 8) has been subtracted from each matrix element. A Gaussian smoothing filter with kernel size of $\sim 50$ px was applied to the spectral dimensions of Figure 10 to reduce noise, see Section 3. The intrinsic response variations affect the modulated solar intensities acquired during flight, thus a pixel-to-pixel demodulation strategy is required. The later is justified because the error that is introduced by demodulating using the spectrally averaged matrix, which can be estimated by $|\Delta X_{\mathrm{sp\_avg}}|=|\hat{O}^{-1}_{\mathrm{sp\_avg}}\hat{O}- \mathbf{1}|$, is larger than $|\Delta X_{{\mathrm{max}}}|$ at many of the measured wavelengths, see Figure 11. Note that, such a pixel-wise demodulation implies that any morphological transformation done to the solar intensities before demodulation must be also applied to the corresponding demodulation matrix. This is particularly relevant for SUSI, because the planned data reduction strategy implies correcting for major instrumental effects and applying numeric image restoration (see van Noort 2017 and Section 2.1) to the solar measurements before demodulating. The instrumental corrections include, among others, flat fielding and reduction of the spectrograph smile distortion, see Hoelken et al. (2024) for extra details. The smile correction involves a morphological deformation that must be also implemented to the corresponding modulation matrix.

**Figure 12:**
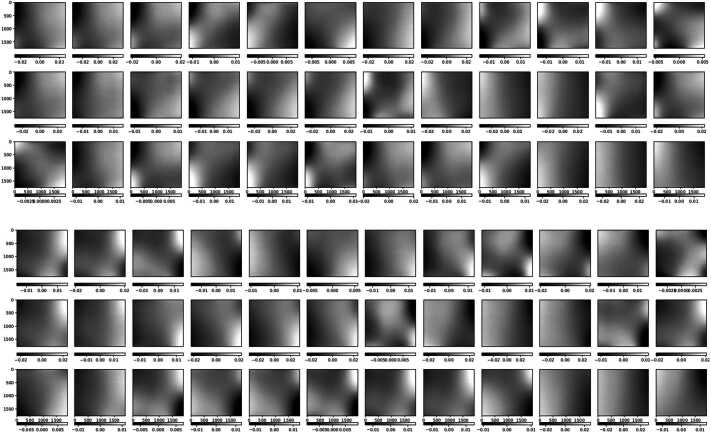
Same as Figure 10, except that a $100\times 100$ px Gaussian smoothing filter has been applied to reduce fringes.

**Figure 13:**
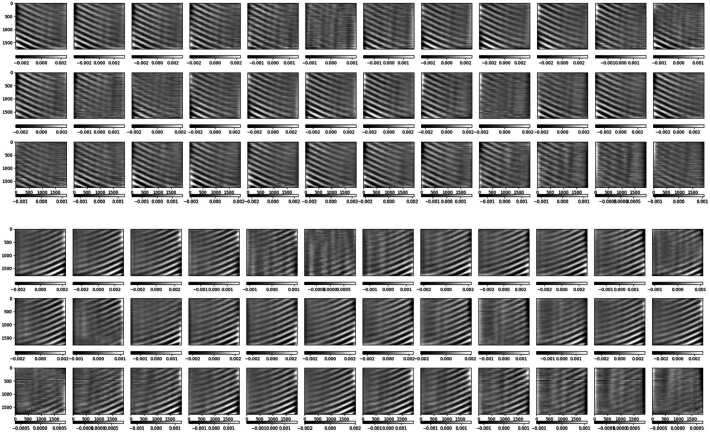
Difference between the matrices of Figures 10 and 12.

**Figure 14:**
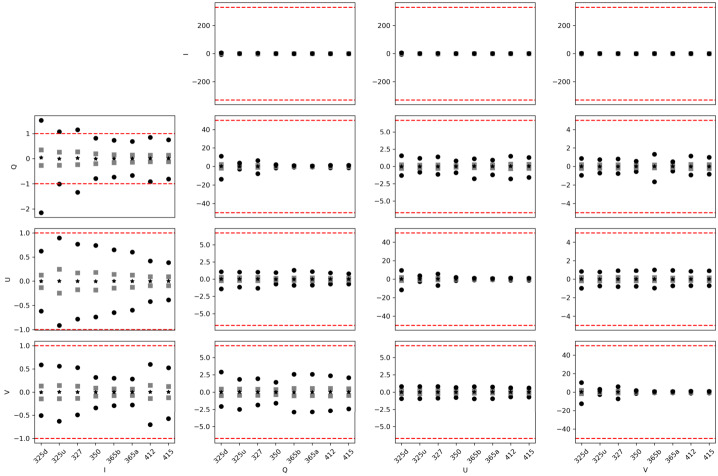
Estimated crosstalk that would be produced by interference fringes of the modulation matrices if left uncorrected. We show the percentiles 99 and 1 (*black circles*), 25 and 75 (*gray squares*) and the median (*black asterisk*) for each element of $|\Delta X_{\mathrm{fringes}}|$ and each calibration measurement (*horizontal axes*) of the SP1 channel. The *red lines* denote the limits defined by $|\Delta X_{{\mathrm{max}}}|$. The figures for SP2 are practically identical.

The third effect, also clearly visible in Figure 10, is optical interference fringes. For the fringes to be visible in the modulation matrix, they must be either polarized or variable with time, see e.g., Semel (2003), McCall, Hodgkinson, and Wu (2015). Because the dominant fringes observed in Figure 10 are not present in images acquired during hang tests (see Korpi-Lagg et al. 2025) using solar light, we attribute their source to the external optical elements used during the calibration measurements. These include the PSG, LED light source and related feeding lenses, which did not include anti-reflective coatings optimized for the NUV spectral range. Therefore, these fringes must be corrected in the modulation matrix before utilizing it to demodulate solar images. This can be done using a variety of techniques, e.g., Gaussian filtering, Fourier filtering, sinusoidal fitting, etc., because the fringes period is shorter that the size of the intrinsic matrix variations across the sensor. For example, Figure 12 show the effect of applying a $100\times 100$ px Gaussian smoothing filter to reduce fringes. The difference matrix of Figure 13 added to $O_{\mathrm{sp\_avg}}$, gives a $O_{\mathrm{fringes}}$ that can be used to estimate the crosstalk produced by fringes if left uncorrected, namely $|\Delta X_{\mathrm{fringes}}|=|\hat{O}^{-1}_{\mathrm{fringes}}\hat{O}_{\mathrm{sp \_avg}}-\mathbf{1}|$, see Figure 14. Correcting any other fringe present in the modulated intensity images acquired during flight has to be done independently and is more difficult, due to the large span of spatial frequencies of solar structures. Considerable fringes correction can be achieved by using flat fielding data acquired close in time during flight, as demonstrated for SUSI by Hoelken et al. (2024). We note that the final fringes removal strategy will be selected based on its performance quantified using reference unpolarized data acquired during flight, see Section 3.3.3.

#### Residual Crosstalk Correction

**Figure 15:**
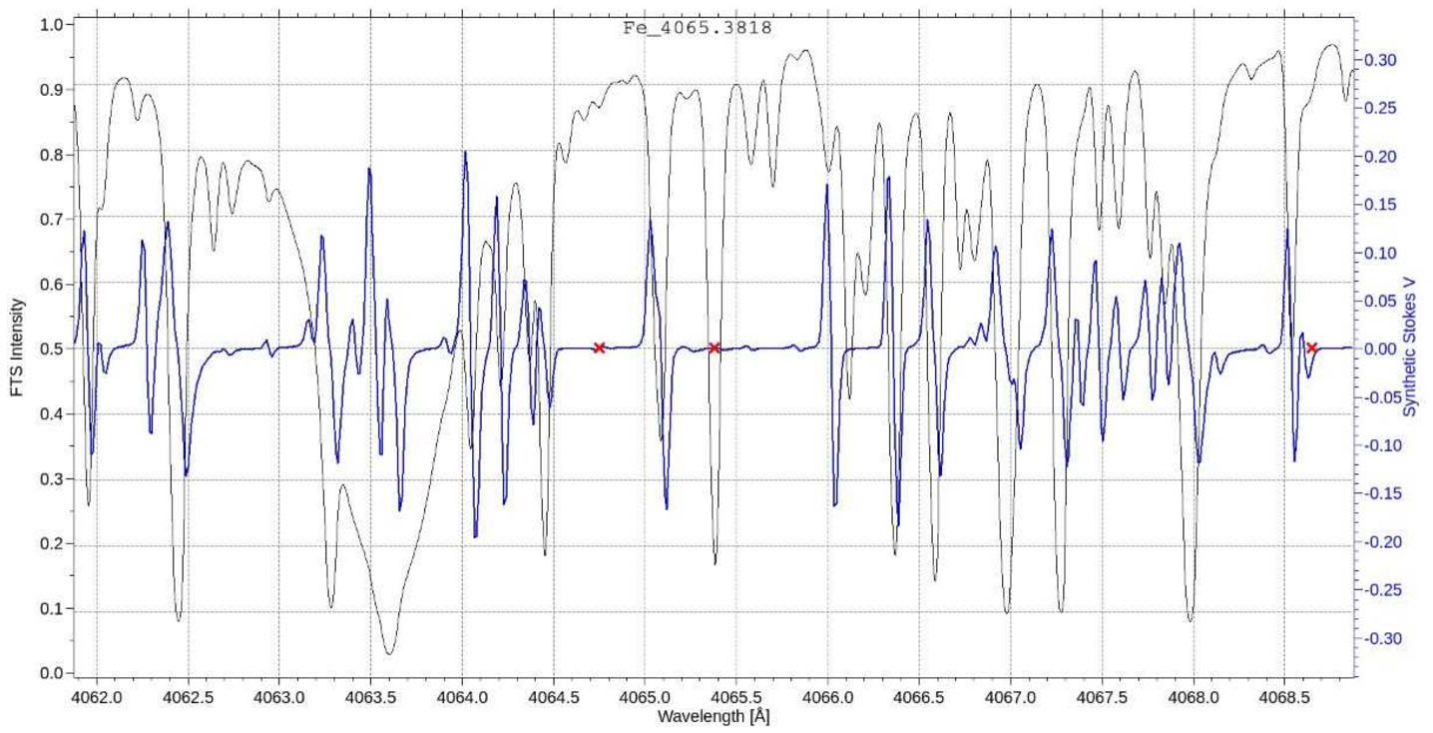
Spectral region around the Fe I line at 406.538 nm (see the *central red cross*). This line presents no obvious blend with other nearby deep lines, as seen in the FTS atlas intensity in black (ref. to the left vertical axis) and a zero effective Landé g-factor. This line was observed for various solar targets as a zero-polarization reference to assist in SUSI data reduction. Note the very low estimated Stokes V amplitude in blue (ref. to the right vertical axis), obtained from a numerical simulation with 1 kG magnetic field. See the text for extra details.

Residual crosstalk can be further reduced during data reduction in an ad hoc fashion, using observations of targets with known polarization properties. These properties include negligible polarization levels, large spectral symmetry of the Zemman polarization profiles, and low spatial correlation between Stokes images. For example, the $I\to Q,U,V$ crosstalk highlighted in bold in Section 3.3 can be reduced by minimizing $I$ to $Q$, $U$ and $V$ correlation, which also minimizes the polarization of null targets such as solar continuum (see below). This is used in different techniques, such as Sanchez Almeida and Lites (1992), Kuhn et al. (1994), Schlichenmaier and Collados (2002), Collados (2003), Jaeggli et al. (2022). Telescope polarization can also be addressed using very low polarization targets, see the work by Derks, Beck, and Martínez Pillet (2018), based on low magnetic sensitivity spectral lines. With SUSI, we have acquired the following zero-polarization observations to help reducing residual $I\to Q,U,V$ calibration crosstalks and other instrumental effects, such as jitter or residual atmospheric seeing induced crosstalks (see e.g., Iglesias et al. 2016):

- Solar continuum: We acquired both fixed-slit and scans of different speeds (see Korpi-Lagg et al. 2025) at disk center, where the Solar continuum wavelengths are known to be unpolarized (Kemp 1970).
- Quiet Sun: Some of the measurements mentioned above are acquired at quiet regions of the Sun (showing no prominent magnetic activity) and last for hours. Observations that are averaged in time for a duration much longer that the typical solar granulation lifetime, $\approx 9$ min (Bahng and Schwarzschild 1961), will exhibit polarization levels that are lower than SUSI’s target sensitivity in multiple spectral lines.
- Magnetically insensitive spectral lines: We acquired observations of solar targets with various Stokes I contrasts levels (quiet Sun, Sunspots, etc.) at the $406.0-408.6$ nm spectral window. This window includes the Fe I absorption line at 406.538 nm, which has a zero effective Lande g-factor, according to the Vienna Atomic Line Database (Ryabchikova et al. 2015), and no obvious blends with other nearby magnetically sensitive lines, according to the Fourier Transform Spectrometer (FTS) solar atlas (Stenflo 2015). This photospheric line has been used in the past to study Solar super-granulation and should exhibit no intrinsic polarization signals. The expected Stokes V signal obtained from a numeric simulation assuming a 1 kG magnetic field is shown in Figure 15, see Riethmüller and Solanki (2019) for extra details on the simulation.

Residual crosstalks between $Q$, $U$ and $V$ are typically reduced by minimizing their spatial cross-correlation. To this end, Solar observations with non-zero polarization levels, such as those acquired by the nominal SUSI science program, are preferred, see Jaeggli et al. (2022) for a review of different techniques.

## Summary and Outlook

SUSI is designed to achieve high polarimetric accuracy in a broad spectral range (309 – 417 nm), using a rotating-waveplate-based PMU, a PBS cube and two custom-made SP cameras working in strict synchronization. All raw camera frames recording the modulated intensities are stored on-board during the flight of Sunrise, with high cadence (256 ms or 12 frames per modulation cycle) and a small spectral (10 – 16 mÅ) and spatial (0.03 arcsec) sampling, to allow for the greatest possible flexibility during post-flight data processing. The polarization demodulation is done during data reduction to retrieve the full input Stokes vector using the demodulation matrices obtained with the pre-flight polarimetric calibration.

The calibration procedure involves using LED light sources for different wavelength ranges, and a motorized PSG to generate 40 well-defined calibration Stokes parameters that are measured with SUSI. The measurements are used to fit wavelength- and field-dependent SUSI modulation matrices, along with additional unknowns: PSG waveplate retardance and position angle, and input intensity scaling coefficients. Given that the CMOS sensor used in the cameras has a rolling shutter, each sensor row samples a different PMU rotation angle, and thus an independent fit is done for each pixel in the FOV of each SP camera. We performed 8 standalone calibration measurements at 6 different wavelengths before the integration of SUSI into Sunrise, to assess the instrument’s functionality and its polarimetric response across the spectral working range. The fitted PSG properties are within the expected values and are consistent with the manufacturer specifications for the PSG waveplate. The fitted modulation matrices vary across SUSI FOV, which demands for a pixel-to-pixel demodulation strategy. The matrices also present wavelength-dependent optical interference fringes, most likely produced by the slit plate. Since the fringes spatial period is considerably shorter than the scales of other modulation matrix variations observed across the FOV, filtering, e.g. with a low pass, is unproblematic. The optimal fringes correction technique to be used consistently in both polarimetric calibration and scientific data, will be selected based on its performance in zero polarization observations.

On the other hand, the repeatability measured in standalone calibrations satisfies the error requirements to reach a polarimetric sensitivity of $1\times 10^{-3}$ in the targeted observing regime. Some $I\to Q,U,V$ elements exceeding the error requirements can be calibrated post-facto, by using the zero polarization observations acquired during flight, including that of the magnetically insensitive Fe I 406.538 nm photospheric line. Finally, the measured polarimetric efficiencies are within $\sim 10\%$ of the design values, with the reflected PBS channel (SP2 camera) presenting up to $\sim 20\%$ lower efficiencies in Q, U and V.

In order to retrieve accurate solar atmospheric quantities, the polarimetric response of the full Sunrise beam path, from entrance aperture to SUSI SP cameras, must be calibrated. We have performed additional polarimetric calibrations at the F1 and F2 focal positions within the Sunrise beam path which are currently under analysis. Their results are to be combined with the standalone SUSI calibration presented here, in order to derive and validate the final calibration methodology to be applied to SUSI scientific data. Such an analysis will be reported in a follow-up paper, including a validation using solar observations acquired both during pre-flight ground tests with Sun pointing, and during the Sunrise scientific flight.

## Data Availability

No datasets were generated or analysed during the current study.
